# Comparative Analysis of Major Mosquito Vectors Response to Seed-Derived Essential Oil and Seed Pod-Derived Extract from *Acacia nilotica*

**DOI:** 10.3390/ijerph15020388

**Published:** 2018-02-23

**Authors:** Perumal Vivekanandhan, Raji Venkatesan, Govindaraju Ramkumar, Sengodan Karthi, Sengottayan Senthil-Nathan, Muthugoundar Subramanian Shivakumar

**Affiliations:** 1 Molecular Entomology Laboratory, Department of Biotechnology, School of Biosciences, Periyar University, Salem, Tamil Nadu 636 011, India; mosqvk@gmail.com (P.V.); venkatesan747@gmail.com (R.V.); ayvidram@gmail.com (G.R.); skentomology@gmail.com (M.S.S.); 2 Division of Biopesticides and Environmental Toxicology, Sri Paramakalyani Centre for Excellence in Environmental Sciences, Manonmaniam Sundaranar University, Alwarkurichi, Tirunelveli, Tamil Nadu 627 412, India; karthientomology@gmail.com

**Keywords:** *Acacia nilotica*, seed essential oil, crude extracts, bioassay, larvicidal activity, smoke toxicity and mosquitoes

## Abstract

Botanical metabolites are increasingly realized as potential replacements to chemical insecticides. In the present study, *Acacia nilotica* seed essential oil and seed pod solvent extracts were tested for bioefficacy against three important types of mosquitoes. Mortality was recorded 24 h post-treatment, while smoke toxicity of adult mosquitoes was recorded at 10 min intervals for 40 min. Seed pod powder was extracted with different solvents and hydrodistilled seed oil chemical constituents were determined by using Gas chromatography mass spectroscopy (GC-MS) -. Larvicidal and adulticidal efficacy of seed hydrodistilled essential oil and solvent extracts were tested against larval and adult mosquitoes. The seed hydrodistilled oil provided strong larvicidal activity against *Anopheles stephensi*, (LC50 (lethal concentration that kills 50% of the exposed larvae) = 5.239, LC90 (lethal concentration that kills 90% of the exposed larvae) = 9.713 mg/L); *Aedes aegypti*, (LC50 = 3.174, LC90 = 11.739 mg/L); and *Culex quinquefasciatus*, (LC50 = 4.112, LC90 = 12.325 mg/L). Smoke toxicities were 82% in *Cx. quinquefasciatus*, 90% in *Ae. aegypti*, and 80% mortality in *An. stephensi* adults, whereas 100% mortality was recorded for commercial mosquito coil. The GC-MS profile of seed essential oil from *A. nilotica* showed the presence of hexadecane (18.440%) and heptacosane (15.914%), which are the main and active compounds, and which may be involved in insecticidal activity. Overall findings suggest that the seed oil showed strong mosquitocidal activity against mosquito vectors and therefore may provide an ecofriendly replacement to chemical insecticides.

## 1. Introduction

Mosquitoes are vectors of many tropical and subtropical pathogens that may cause severe health problems in countries where they are endemic [1]. Specifically, *Anopheles* species vector malaria worldwide. *Aedes aegypti* and *Ae. albopictus* are primary carriers for viruses that cause dengue fever, dengue hemorrhagic fever, chikungunya fever, yellow fever, and Zika [2,3]. *Culex quinquefasciatus*, a vector of lymphatic filariasis, is widely distributed in tropical regions [4]. Lymphatic filariasis is possibly the most rapidly spreading mosquito vector-borne disease to humans in many part of the world and, worldwide, 146 million people suffer from lymphatic filariasis transmitted by *Cx. quinquefasciatus* [5]. Chemical pyrethroids have been relied upon as the insecticides of choice for mosquito control, due to their low mammalian toxicity and quick knockdown activity. In many parts of the world, mosquito vectors have developed resistance against the synthetic chemical insecticides, thus rendering their use for control in some areas ineffective [6,7,8,9]. 

Nature provides several bioactive compounds for the control of mosquitoes, namely plant-derived products, marine algal-derived products, microbial-derived products, and other biological compounds. The use of biological-derived products of plant origin is recommended because they are effective, environmentally friendly, and generally have a low toxicity [10]. Plant oils have rich sources of secondary bioactive metabolites that can be obtained from the nonwoody parts, particularly leaves and seeds through steam distillation or hydrodistillation. Essential oils are rich in species belonging to Rutaceae, Asteraceae, Umbelliferae, Myrtaceae, and Lamiaceae families [11]. The plant-derived oils have biological activities, namely antibacterial, antifungal, antifeedant, as well as insecticidal activities [12,13]. Plant-based essential oil and crude extract have strong ovicidal activity against mosquito eggs [14] and larvicidal activity [15,16,17]. Biotoxicity of neem cake extract shows strong insecticidal activity against mosquitoes [18]. Many research studies have evaluated the use and efficacy of natural plant products against arthropod pests [14,15,16,17,18,19,20,21,22]. A previous report showed that *Coriandrum sativum* (Apiaceae) essential oil shows strong insecticidal activity against *A. albopictus* (larvae LC_50_ was 421 ppm and LC_90_ was 531 ppm)[15]. Dhanasekaran et al. [23] also reported that actinobacterial extract shows remarkable larvicidal activity against mosquitoes. *Cassia fistula* L flower ethyl acetate crude extract shows strong pesticidal activity against Lepidopteran pests [24].

Several laboratory evaluations have reported the use of plant secondary metabolites and essential oils against larval and adult mosquitoes [12,25,26,27]. *Vachellia nilotica*, widely known as *Acacia nilotica*, belongs to the Fabaceae family, and is present in India and other parts of the world. This plant is used as medicine for humans and animals. Chronic larval toxicity from *A. nilotica* stem bark acetone extracts against major mosquito species has been reported by Chaubal et al. [28]. Acute toxicity of acetone leaf extracts of *A. nilotica* at 212.1 mg/L and chronic toxicity at 144.2 mg/L was observed in larval *Cx. pipiens*; in addition, these extracts have shown the ability to suppress egg hatchability and adult emergence [29].

Commercial available plant-derived essential oils and secondary metabolites are very effective as for good repellents and their insecticidal agents can be incorporated into integrated vector control approaches [30]. *A. nilotica* seed pods are reported to contain saponins, terpenes, and tannins, all of which display antiparasitic activity [31]. Although *A. nilotica* crude extracts have insecticidal activity [29,31], there are no reports of seed pod extracts and seed essential oils being used as mosquito control agents. Therefore, the objective of our study was to investigate the mosquitocidal activity of seed oil and seed pod solvent extracts of *A. nilotica* against three important mosquito vectors. 

## 2. Materials and Methods

### 2.1. Collection of Plant Materials

**Figure 1:**
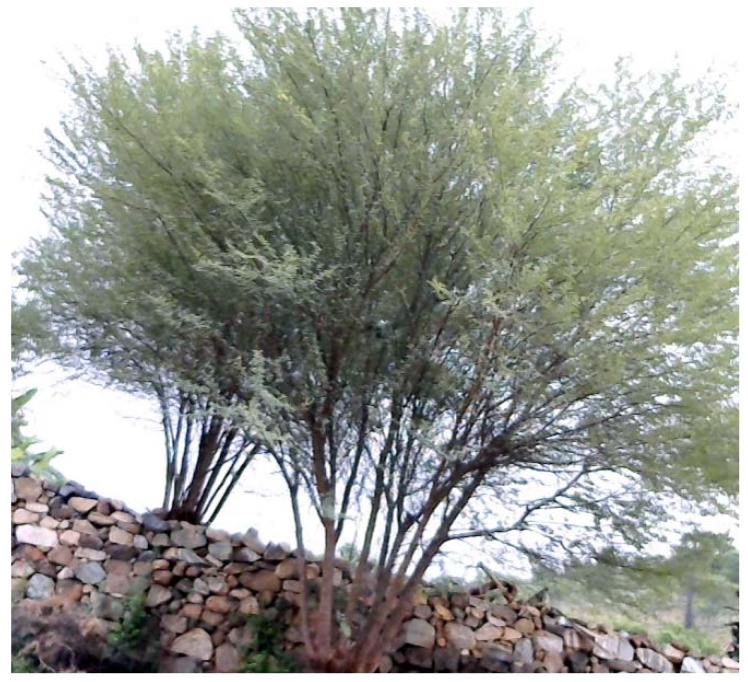
*Acacia nilotica*.

*A. nilotica* (Figure 1) plant seeds were collected from the grounds of Periyar University campus in Tamil Nadu, India (11.7122° N, 78.0709° E) on 15 January 2015. Specimens were deposited in the laboratory (the university herbarium, Salem, Tamil Nadu, India) as voucher no-28.

### 2.2. Preparation of Plant Extracts

The seed pods were shade dried for 7–10 days. The dried seed pods were powdered mechanically using a commercial electrical stainless steel blender and the powdered seed pod (200 g) was extracted with hexane (350 mL), petroleum benzene (350 mL), chloroform (350 mL), ethyl acetate (350 mL), and acetone (350 mL) in a Soxhlet apparatus (boiling point 60–85 °C) for 7 h. Finally, the crude extract was concentrated to reduce the solvent content by using a rotary evaporator (Superfit, Mumbai, India, Model-R/150/01) under reduced pressure 23–27 mm Hg at 48 °C, with the resultant residue stored at room temperature.

### 2.3. Hydrodistillation of Essential Oils

Oils from the powdered seeds were extracted by hydrodistillation using a modified Clevenger apparatus by adopting method from Dua et al. [32]. In each case, 20 g of seed powder was distilled in 300 mL of distilled water in a 500 mL flask for 2 h. The entire sample of seed pod essential oils was extracted and collected in sanitized glass vials. Anhydrous sodium sulfate was used to remove water traces, and the *A. nilotica* seed pod essential oil samples were preserved at 4 °C for further experiments.

### 2.4. Maintenance of Mosquito Larvae

Insecticide-susceptible mosquito culture was provided by IVCZ, Banahalli, Tamil Nadu, India. Larval cultures were maintained in laboratory conditions. All evaluations were carried out at laboratory temperature condition 27 ± 2 °C and 80–90% Relative Humidity (RH), under a 12:12 photoperiod period. We provided dog biscuits and yeast (3:1 ratio) as the food source for the larvae.

### 2.5. Larval Bioassay

The larvicidal activity of the plant seed pod crude extracts and seed essential oil were tested as per the method recommended by the World Health Organization [33]. Twenty-five fourth instar larvae of each species were transferred to separate plastic cups, in 249 mL of water and 1.0 mL of the desired plant extract and essential oils at different concentrations (10, 50, 150, 200, and 250 mg/L). An equal number of controls were set up simultaneously using tap water. Dead larvae were counted 24 h post-treatment, and the percent mortality was reported from the average of three replicates using formula (1). The LC_50_ (lethal concentration that kills 50% of the exposed larvae) and LC_90_ (lethal concentration that kills 90% of the exposed larvae) values were calculated based on mortality data by using probit analysis according to Abbott’s [34] formula (2).

(1) $$\mathrm{Percentage}\;\mathrm{of}\;\mathrm{mortality}\;=\frac{\mathrm{Number}\;\mathrm{of}\;\mathrm{death}\;\mathrm{larvae}}{\mathrm{Number}\;\mathrm{of}\;\mathrm{larvae}\;\mathrm{introduced}}\;\times \;100$$

(2) $$\mathrm{Corrected}\;\mathrm{percentage}\;\mathrm{of}\;\mathrm{mortality}\;=\;1\;-\;\frac{n\;\mathrm{in}\;T\;\mathrm{after}\;\mathrm{treatment}}{n\;\mathrm{in}\;C\;\mathrm{after}\;\mathrm{treatment}}\;\times \;100$$

where *n* = number of larvae, T = treated, C = control.

### 2.6. Mosquito Coil Preparation

Mosquito coils, for adult biosaays, were prepared by following the method of Vivekanandhan et al. [16] and Ramkumar et al. [35]. The composition of mosquito coils consisted of 5 g of shade dried seed pod powder, 2 g of sawdust, and 2 g of charcoal powder. All ingredients were mixed with distilled water to make a semi-solid condition that changed to 0.5 cm diameter coils. Coils were shade dried in room temperature and used for the knockdown test. Commercial mosquito coils (Good Night, Godrej Consumer Product Lts, Mumbai; allethrin 0.04% *w/w*) were used.

### 2.7. Smoke Knockdown Test

A knockdown test was carried out under laboratory conditions using a mosquito cage (60 cm × 40 cm × 35 cm) [16,35]. Three hundred adult mosquitoes of each species were separately released to the chamber and exposed to the smoke of burning coils for 40 min, with mortality data recorded every 10 mins. The smoke knockdown test was compared with a commercially available coil (Good Night, Godrej Consumer Product Lts, Mumbai. Allethrin 0.04% *w/w*).

### 2.8. Gas Chromatography–Mass Spectrometry (GC–MS)

Clarus 680 was used in the analysis, which employed a fused silica column, packed with Elite-5MS (5% biphenyl 95% dimethylpolysiloxane, 30 m × 0.25 mm ID × 250 μm df), and the components were separated using helium as carrier gas at a constant flow of 1 mL/min. The injector temperature was set at 260 °C during the chromatographic run. One microliter of extract sample was injected into the instrument. The oven temperature was as follows: 60 °C (2 min), followed by 300 °C at the rate of 10 °C min^−1^ and then 300 °C, where it was held for 6 min. The mass detector conditions were: transfer line temperature 240 °C, ion source temperature 240 °C, and ionization mode electron impact at 70 eV, a scan time 0.2 s, and scan interval of 0.1 seconds. Considering fragments from 40 to 600 Da, the spectrums of the components were compared with the database of spectrum of known components stored in the GC-MS NIST (2008) library (mass spectrometry database). The GC-MS analysis was carried out in the Sophisticated Instrument Facility, (SAIF).Vellore Institute of Technology (VIT), Vellore, Tamil Nadu, India. 

### 2.9. Statistical Analysis

Mortality was corrected by using Abbot’s method [34]. Average larval mortality data were subjected to profit analysis for the calculation of LC_50_ and LC_90_ valves and associated 95% confidence limits; chi-square and degrees of freedom (df) were calculated using SPSS-16.00 software(IBM-Corporation, Bengaluru, Karnataka, India).

## 3. Results and Discussion

Botanical products that are locally available and have major roles in mosquito control would lead to many advantages, like offering ecofriendly and cost-effective means to stimulate efforts to protect the public health against disease spreading mosquito vectors [36,37]. A large number of botanically derived essential oils have been extracted and reported for their promising mosquitocidal activity against three important mosquito vectors [3,38]. 

The greatest larvicidal activity was observed in seed essential oil against *An. stephensi*, *Ae. aegypti*, and *Cx. quinquefasciatus*, with LC_50_ and LC_90_ values of 5.239–9.713, 3.174–11.739, and 4.112–12.325 mg/L, respectively (Table 1, Table 2 and Table 3). Ethyl acetate and petroleum benzene seed pod crude extracts provided remarkable larvicidal activity against *An. stephensi*, *Ae. aegypti*, and *Cx. quinquefasciatus*, with LC_50_ and LC_90_ values of 48.326–58.631, 59.122–75.821, and 48.082–65.321 mg/L, respectively (Table 1, Table 2 and Table 3). The results for the seed pod powder smoke toxicity test on adult mosquitoes were as follows: 82% mortality in *Cx. quinquefasciatus*, 90% in *Ae. aegypti*, and 80% in *An. stephensi*, while the commercial coil showed 100% mortality (Table 4). Similar to our study, *Acanthospermum hispidum* leaf crude extract and powder have been shown to have strong mosquitocidal activity on mosquitoes [16]. *Ipomoea cairica* plant oil is also known to have strong larvicidal activity on three major mosquito species [39]. Botanical oils isolated from several different species have shown strong larvicidal activities, for example, oils obtained from *Ocimum gratissimum* showed high larvicidal activity against *An. stephensi* (LC_50_ values 60 to 538 mg/L), and *Lippie sidoides* leaf extract showed notably high larvicidal activity against *Ae. aegypti* (LC_50_: 67 mg/L) [40]. *Cordia leucomalloides* oil shows very strong mosquitocidal activity against *Cx. quinquefasciatus* [7]. Botanically derived oils and their compounds are only highly toxic against targets with treatments where targets have been exposed to higher concentrations for a long time [41]. However, our results show that significant mortality was found in all three major mosquito species. Ajaegbu et al. [42] have reported that plant-derived essential oils show strong mosquitocidal activity against major mosquito species. 

*Lantana camara* essential oil was tested for larvicidal and adult knockdown activity against three major mosquito species [32]. *Tagetes patula* volatile oil has strong mosquitocidal activity with low LC_50_ values 0.0070 mg/cm^2^ against *Cx. pipiens* [43]. Plant-based phytochemicals and plant products are increasingly being used for their efficacy as biocontrol agents for control of insecticide-resistant mosquitoes [44,45]. Biopesticides from plant origin have been shown to be more effective on agricultural and medical pests [45,46]. Nowadays, plant and microbial sources are increasingly used for vector control programs because they have been shown to have the potential to be effective, more target-specific than chemical insecticides, and ecofriendly [8,47,48]. 

Our GC-MS results shows that two major compounds, namely hexadecane (18.440%) and heptacosane (15.914%), may harbor insecticidal potential. Further study on the isolation of hexadecane and heptacosane may be useful in identifying the mosquitocidal potential of these compounds (Table 5). Our findings suggest that *A. nilotica* hydrodistilled seed oil can control mosquito larvae, and mosquito coils developed using *A. nilotica* seed pod powder have toxicity against mosquitoes.

## 4. Conclusions

While the development and use of botanically derived compounds can be cost effective in developing countries, there are still may issues which need to be addressed in order to allow for large-scale production. The current results clearly show that hydrodistilled essential oil from *A. nilotica* seeds has strong mosquitocidal activity against major mosquito species. *A. nilotica* seed hydrodistilled essential oil harbors bioactive molecules like hexadecane and heptacosane, which can be isolated and further tested for effective mosquitocidal activity. Future evaluation on the separation of the active principles responsible for the mosquitocidal activity can further facilitate the development of ecofriendly plant-based pesticide for controlling mosquito vectors. 

## Figures and Tables

**Table 1:** Larvicidal activity of *A. nilotica* seed extracts and essential oil against the fourth instar larvae of *An. stephensi.*

Mosquito Species	Sample	LC_50_ (LCL–UCL) mg/L	LC_90_ (LCL–UCL) mg/L	df
*An. stephensi*	Acetone	110.00 (108.82–112.11)	171.18 (169.28–174.89)	3
	Ethyl acetate	48.32 (44.11–51.82)	58.63 (56.11–60.82)	3
	Hexane	121.11 (119.96–128.08)	188.88 (186.16–92.82)	3
	Chloroform	118.80 (112.23–120.16)	163.8270 (159.11–165.11)	3
	Petroleum Benzene	51.10 (49.80–55.26)	59.62 (57.62–61.82)	3
	Essential oil *	5.23 * (3.42–6.83)	9.71 * (7.73–10.83)	3

* indicates significantly difference (*p* < 0.01) between different solvent extracts. LC_50_—lethal concentration that kills 50% of the exposed larvae, LC_90_—lethal concentration that kills 90% of the exposed larvae, UCL—upper confidence limit (95%), LCL—lower confidence limit (95%), df—degrees of freedom.

**Table 2:** Larvicidal activities of *A. nilotica* seed extracts and essential oil against the fourth instar larvae of *Ae. aegypti.*

Mosquito Species	Sample	LC_50_ (LCL–UCL) mg/L	LC_90_ (LCL–UCL) mg/L	df
*Ae. aegypti*	Acetone	103.68 (101.11–105.82)	162.03 (159.03–165.81)	3
	Ethyl acetate	59.12 (57.16–61.11)	75.8216 (73.28–79.28)	3
	Hexane	169.25 (165.98–172.90)	201.6231 (200.11–203.82)	3
	Chloroform	158.13 (156.82–160.11)	198.2361 (193.98–203.72)	3
	Petroleum Benzene	45.32 (43.96–47.82)	99.3216 (77.12–81.12)	3
	Essential oil *	3.17 * (2.10–4.67)	11.73 * (9.93–12.90)	3

* indicates significantly difference (*p* < 0.01) between different solvent extracts. LC_50_—lethal concentration that kills 50% of the exposed larvae, LC_90_—lethal concentration that kills 90% of the exposed larvae, UCL—upper confidence limit (95%), LCL—lower confidence limit (95%), df—degrees of freedom.

**Table 3:** Larvicidal activities of *A. nilotica* seed extracts and essential oil against the fourth instar larvae of *Cx. quinquefasciatus*.

Mosquito Species	Sample	LC_50_ (LCL–UCL) mg/L	LC_90_ (LCL–UCL) mg/L	df
*Cx. quinquefasciatus*	Acetone	62.86 (59.32–64)	90.80 (83.28–91.99)	3
	Ethyl acetate	61.9861 (59.63–63.11)	73.26 (71.71–75.11)	3
	Hexane	152.36 (147.82–161.32)	201.11 (200.86–203.60)	3
	Chloroform	116.00 (114.11–117.02)	186.13 (185.01–190.11)	3
	Petroleum Benzene	48.08 (47.96–50.62)	65.32 (63.06–67.162)	3
	Essential oil *	4.11 * (3.23–5.44)	12.32 * (11.32–14.01)	3

*indicates significantly difference (*p* < 0.01) between different solvent extracts. LC_50_—lethal concentration that kills 50% of the exposed larvae, LC_90_—lethal concentration that kills 90% of the exposed larvae, UCL—upper confidence limit (95%), LCL—lower confidence limit (95%), df—degrees of freedom.

**Table 4:** Smoke toxicity of *A*. *nilotica* plant seeds powder mosquito coil compared with commercial mosquito coil and mosquito coil without any plant powder on major mosquito vectors.

Mosquito Species	Observation (in Minutes)	% Mortality from *A. nilotica* Powder Mosquitoes Coil	% Mortality from Commercial Mosquito Coil	% Control Mortality
*Cx. quinquefasciatus*	10	00	00	0
	20	20	10	1
	30	28	50	1
	40	82	100	0
*Ae. aegypti*	10	0	0	0
	20	0	10	1
	30	10	50	1
	40	90	100	0
*An. stephensi*	10	3	0	1
	20	12	18	0
	30	28	50	0
	40	80	100	1

**Table 5:** Chemical constituents GC-MS analysis of *A. nilotica* seed essential oil.

S.No	RT ^a^ (min)	Area	Area %	Compound Name	Activity
1	20.330	1,293,815.8	4.394	Hexadecane	Antimicrobial [10]
2	21.131	3,105,857.5	10.548	Sulfurous acid, butyl tridecyl ester	Antioxidant [11]
3	21.911	4,685,758.0	15.914	Heptacosane	No reports
4	22.331	3,055,476.2	10.377	1,2-Benzenedicarboxylic acid, isodecyl octyl ester	Antimicrobial and antifouling [27]

^a^ RT-Retention Time; S. No-Serial Number.
